# Mini Nutritional Assessment Scores Indicate Higher Risk for Prospective Mortality and Contrasting Correlation With Age-Related Epigenetic Biomarkers

**DOI:** 10.3389/fendo.2019.00672

**Published:** 2019-10-01

**Authors:** Alberto Montesanto, Patrizia D'Aquila, Veronica Rossano, Giuseppe Passarino, Dina Bellizzi

**Affiliations:** Department of Biology, Ecology and Earth Sciences (DiBEST), University of Calabria, Rende, Italy

**Keywords:** Mini Nutritional Assessment, epigenetic, biomarkers, DNA methylation, aging, survival

## Abstract

The plasticity of the individual epigenetic landscape that goes to countless rearrangements throughout life is closely the reflection of environmental factors such as chemical exposure, socio-economic status and nutrient intakes both early and late in life. The Mini Nutritional Assessment (MNA) is a well-validated tool for assessing malnutrition in old people. It includes 6 (MNA-SF) or 18 (MNA-LF) self-reported questions derived from general, anthropometric, dietary, and self- assessment. We evaluated the association between the nutritional status, as measured by MNA, and methylation biomarkers we previously demonstrated to be associated with chronological and biological age in human. We found that malnutrition is positively correlated with DNA methylation status at the global level, in line with our previous reports. On the contrary, most of the sites located within specific genes, which were previously reported to be correlated with chronological and biological aging, showed to be not affected by malnutrition, or even to have correlations with malnutrition opposite to those previously reported with frailty. These results may suggest that malnutrition is among the first effects of disability and other age- related problems and a generalized non-specific epigenetic remodeling may be the initial response of the organism. By contrast, the fine remodeling of specific genomic sites is scarcely affected by malnutrition and may respond to a more complex interaction of different factors. Therefore, although malnutrition in the elderly is certainly a risk factor for survival, this is partially independent of the aging process of the organism which leads to the methylation remodeling previously described to measure chronological and biological aging.

## Introduction

The multi-causal phenotypic variability among elderly individuals has led researchers to evaluate changes in a wide variety of biological parameters in order to have a more comprehensive insight of health at old age and, possibly, predict both the quality of aging and the onset of age-related diseases (1, 2). In this context, in addition to non-directional changes in DNA methylation patterns, referred to as epigenetic drift, directional, and non-stochastic hyper- or hypo-methylation events, occurring over time at discrete CpG sites throughout the genome, turned out to be very useful, and also able to be predictive of both chronological and biological aging (3–7). These age-Differentially Methylated Regions (a-DMRs) have been found associated with survival chance, disability, frailty, multi-morbidity, thus determining the overall variation in life expectancy (8–12). What is more, the setup of a series of multi-tissue age estimator models, namely epigenetic clocks, is allowing the prediction of all-cause mortality independent of several risk factors (13–24).

Considered their function as substrates or cofactors for epigenetic enzymes, nutrients and their metabolites are the main environmental factors able to modify the epigenetic landscape as well as health and life expectancy throughout the lifetime (25–27). Dietary manipulation of methyl donors (either supplementation or deficiency) as well as calorie restriction (CR) have been recognized as responsible for global rearrangements of DNA methylation profiles (28–33). Furthermore, according to the “developmental origins of health and disease” hypothesis, already *in utero*, energy-rich, protein-deficient, micronutrient-deficient, and/or methyl donor-rich diets induce multi-tissue perturbations of methylation profile in mothers (34). These perturbations can be transmitted to next generation thus regulating in offspring long term metabolic processes and contributing to age phenotypes and age-related diseases (26, 29, 35–39). With aging, changes or loss in appetite, mainly due to the reduction of acuity in sense organs, in the secretion of hunger hormones, gastrointestinal motility as well as the inability in preparing food, depression, and dementia, lead to a significant reduction of food intake (40–42). Consequently, a loss in weight, muscle mass, strength, and physical function is generally observed and it is retained to be the main origin of the weakness and the decline in functional ability, including conditions such as sarcopenia and frailty (43–48). On the contrary, appropriate nutritional status was held responsible for the safeguard of healthy aging and linked to favorable outcomes in health, by retarding the detrimental consequences of aging and the prevention/treatment of a variety of diseases (44, 48–53).

Taking into account the relationship among nutritional status, DNA methylation patterns, and biological aging, we evaluated the association between nutritional status as measured by the Mini Nutritional Assessment (MNA), a validated assessment tool to measure nutrition status in elderly people, and previously identified age-related methylation biomarkers at both nuclear and mitochondrial level.

## Materials and Methods

### Study Population

Socio-demographic characteristics of the population sample analyzed in this study were previously reported (54). Briefly, the sample included 302 subjects living in Calabria (South Italy) subdivided into two groups: the first (S1) comprised 191 subjects younger than 85 years (101 women and 90 men, mean age 73.0 years), the second (S2), 111 subjects aged 85 or over (54 women and 57 men, mean age 97.1 years). Samples were collected within the framework of several recruitment campaigns carried out for monitoring the quality of aging in the whole Calabria region from 2002 onwards. Subjects older than 90 years were identified through the population registers and then contacted by specialized personnel and invited to join the study.

Younger subjects were contacted through general physicians. Finally, each subject was recruited after a complete multidimensional geriatric assessment with detailed clinical history, including anthropometric measures and a set of the most common tests to assess cognitive functioning, functional activity, physical performance, and depression. In addition, common clinical hematological tests were performed. White blood cells (WBC) from blood buffy coats were used as source of DNA.

Vital status at near 9 years from the baseline visit was traced for 189 subjects (98.95%) in S1 and for all the 111 subjects (100%) in S2 through the population registers of the municipalities where the respondents lived.

### Mini Nutritional Assessment

The Mini Nutritional Assessment is a well-validated tool for assessing malnutrition in old people (55). It includes 18 self-reported questions derived from general, anthropometric, dietary, and self- assessment. In particular, the short form of the MNA (MNA-SF) is a screening tool consisting of six questions on food intake, weight loss, mobility, psychological stress, or acute disease, the presence of dementia or depression, and body mass index (BMI). The maximum score for this part is equal to 14. A score equal to or higher than 12 indicates that the subject under study has an acceptable nutritional status thus excluding malnutrition and/or malnutrition risk, meanwhile, a score ≤ 11 implicates to proceed with the complete version of the MNA (MNA-LF) (56). This version consists of 12 additional items and provides a maximum possible overall assessment of 30 scores: a score of fewer than 17 indicates malnutrition, a score of 17–23.5 indicates a risk for malnutrition and a score higher 23.5 indicates well-nourishment (57).

### Epigenetic Biomarkers

For the assessment of the correlation between MNA and the epigenetic status of biomarkers, we focused on DNA methylation of CpG sites, located within both nuclear and mitochondrial genes, we previously found associated with chronological and biological aging: one CpG site located within the human mitochondrial 12S ribosomal RNA (*MT-RNR1*) gene, 4 CpG units (CpG_5, CpG_18.19, CpG_23.24, CpG_25.26) falling into the promoter of the ribosomal RNA genes (rDNA), and 12 CpG sites (BNIP3L_Amplicon1_CpG_10, COX18_CpG_2, COX18_CpG_15, GABARAP_Amplicon2_CpG_7.8, MARCH5_Amplicon1_CpG_2.3.4, RAB32_Amplicon1_CpG_24, RHOT2_Amplicon1_CpG16, TFB1M_CpG_12.13) located within promoter regions of genes involved in mitochondrial quality control (10, 11, 58) (Table 1; Figure S1).

**Table 1:** DNA methylation levels of biomarkers evaluated in the population sample.

**Gene name**	**CpG site name**	**Chromosome localization**	**Methylation levels (mean and SD)**
Mitochondrially encoded 12S rRNA	MT-RNR1	MT: 932	37.1 ± 26.7
Ribosomal RNA	rDNA_CpG_5	21:8205862	21.0 ± 12.2
	rDNA_CpG_18.19	21: 8205929, 8205935	34.0 ± 14.4
	rDNA_CpG_23.24	21: 8205976, 8205979	34.0 ± 14.4
	rDNA_CpG_25.26	21: 8205995, 8206008	21.0 ± 14.7
BCL2 interacting protein 3 like	BNIP3L_Amplicon1_CpG_10	8: 26383187	45.7 ± 18.2
Cytochrome c oxidase assembly factor COX18	COX18_CpG_2	4: 73069369	10.6 ± 4.4
	COX18_CpG_15	4: 73069484	1.8 ± 2.6
GABA type A receptor-associated protein	GABARAP_Amplicon2_CpG_7.8	17: 7242835, 7242839	1.6 ± 2.0
Membrane associated ring-CH-type finger 5	MARCH5_Amplicon1_CpG_2.3.4	10: 92290747, 92290750, 92290759	2.9 ± 1.8
RAB32, member RAS oncogene family	RAB32_Amplicon1_CpG_24	6: 146543728	9.8 ± 5.6
Ras homolog family member T2	RHOT2_Amplicon1_CpG_16	16: 668822	9.2 ± 5.5
Transcription factor B1, mitochondrial	TFB1M_CpG_12.13	6: 155314493, 155314495	40.6 ± 7.9

*Gene name, CpG site name, and chromosome position are reported*.

In addition, we had also considered the association of MNA and the overall degree of methylation of the human genome as measured by Global DNA Methylation Index (GDMI) (8).

### Analytic Approach

We compared S1 and S2 groups with regard to study variables and covariates. We used the unpaired *t*-test for continuous variables and chi-square for categorical ones.

For each group, Kaplan-Meier survival curves were estimated for the MNA risk categories (MNA-SF < 12 and MNA-LF < 17 or MNA-LF < 23.5). In order to evaluate their predictive values with respect to mortality risk, the obtained survival curves were then compared by log-rank test. Subjects alive after the follow-up time were considered as censored, and this time was used as the censoring date in the survival analyses. In addition, Hazard ratios (HR) and 95% Confidence Intervals (95% CI) were estimated by using Cox proportional hazard models taking also into account possible confounder variables (age and gender).

A linear regression model was used to assess the association between the variability of epigenetic markers and MNA scores. Analyses were adjusted for age at the recruitment and gender.

All statistical analyses were performed using IBM SPSS statistics for Windows, V.25 (IBM Corp).

## Results

### MNA Assessment

Subjects younger and older 85 years of age were included in the study. Table 2 shows the demographic characteristics of the analyzed sample and the nutritional status of the elderly as resulting by MNA assessment. Out of the 302 subjects we analyzed, 215 (71.2%) exhibited an adequate nutritional status scoring of 12 or more. In particular, this accounted for 79.6% of subjects younger than 85 years and 56.8% of older subjects. On the contrary, 87 subjects (28.8%) had a score lower than 12 and went through the complete assessment of MNA (MNA-LF). After the complete test, in S1 group, about 10% turned out to be malnourished, 64.1% at risk, while the remaining ones were well-nourished (25.6%). In S2 group we found a near-tripled proportion of malnourished subjects (27.1%) with respect to S1, a similar proportion of individuals at risk for malnutrition (about 65%), and a reduced number of well-nourished subjects (8.3%).

**Table 2:** MNA risk categories in the analyzed sample by age-group.

		**Age group**	
		**S1 group (≤85 years)**	**S2 group (>85 years)**
MNA-SF score	<12	39 (20.4%)	48 (43.2%)
	≥12	152 (79.6%)	63 (56.8%)
MNA-LF score	<17	4 (10.3%)	13 (27.1%)
	17-23.5	25 (64.1%)	31 (64.6%)
	≥24	10 (25.6%)	4 (8.3%)

### MNA Scores and Survival

**Figure 1:**
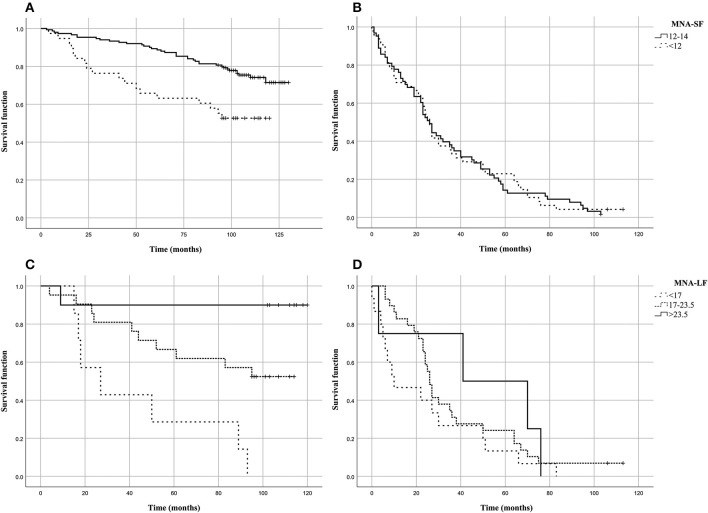
Kaplan–Meier estimates of the survival functions in people with malnutrition (MNA-SF<12 or MNA-LF<17) vs. those that did not show malnutrition in the analyzed sample. **(A)** MNA-SF in S1 group; **(B)** MNA-SF in S2 group; **(C)** MNA-LF in S1 group; **(D)** MNA-LF in S2 group.

Figure 1 shows the Kaplan–Meier estimates of the survival functions in people with malnutrition (MNA-SF < 12 or MNA-LF < 17) vs. those that did not show malnutrition in the S1 and S2 groups of the analyzed sample. We found that in S1 group people with malnutrition (MNA-SF < 12, MNA-LF < 17) lived shorter than people that did not show malnutrition, while in S2 group no association was found between malnutrition and mortality risk (Figures 1B,D). In fact, in S1 group after the follow-up period, 48.7% of those who were at risk for malnutrition (MNA-SF < 12) in S1 group had died (panel A), compared to 25.8% of those who were not a risk at baseline (*P* = 0.001). Among the subjects who obtained a screening score lower than 12 in S1 group, we found that after the follow-up period, all subjects with severe malnutrition (MNA-LF < 17) died (panel C), compared to 56.0% of those who were at risk for malnutrition (MNA-LF > 17 and MNA-LF < 23.5) and 10.0% of those who were not at risk (MNA-LF>23.5) (*P* = 0.002).

To assess the independent predictive validity of malnutrition in terms of survival in S1 group, we evaluated its association with prospective mortality risk by Cox proportional hazard models. After adjusting for age at recruitment and gender the association between MNA-SF and mortality risk did not hold statistical significance (HR = 1.518, 95% CI: 0.828–2.785, *P* = 0.177). As it regards MNA-LF, we found that malnourished subjects (MNA-LF < 17) had a significantly increased risk of mortality with respect to subjects with a normal nutritional status (HR = 17.6, 95% CI = 1.583–195.588, *P* = 0.020) as well as those with a nutritional status at risk (HR = 5.854, 95% CI = 0.733–46.726, *P* = 0.095), also after adjusting for age and gender.

### MNA Scores and Epigenetic Markers

The analysis of the relationship between nutritional status and epigenetic markers revealed that after adjusting for age at recruitment and gender, GDMI values significantly decreased as MNA-LF scores increased (*P* = 0.052). No significant correlations between MNA-SF and epigenetics biomarkers were detected in S2 group (Table 3).

**Table 3:** Association between MNA-SF and MNA-LF scores and epigenetic markers.

	**MNA-SF**						**MNA-LF**					
	**S1 group**			**S2 group**			**S1 group**			**S2 group**		
	**Beta (SE)**	**T**	***P*-value**	**Beta (SE)**	**T**	***P*-value**	**Beta (SE)**	**T**	***P*-value**	**Beta (SE)**	**T**	***P*-value**
MT-RNR1	0.23 (0.02)	0.20	0.844	−0.65 (1.12)	−0.58	0.562	2.00 (1.28)	1.57	0.127	−1.58 (0.90)	−1.76	0.089
rDNA_CpG_5	**1.35 (0.65)**	**2.06**	**0.042**	−1.89 (1.02)	−1.85	0.074	−0.55 (0.79)	−0.70	0.492	−2.81 (1.35)	−2.09	0.070
rDNA_CpG_18.19	0.75 (0.70)	1.06	0.292	1.17 (1.10)	1.06	0.296	1.18 (1.04)	1.13	0.268	1.27 (1.57)	0.81	0.438
rDNA_CpG_23.24	0.75 (0.70)	1.06	0.292	1.17 (1.10)	1.06	0.296	1.18 (1.04)	1.13	0.268	1.27 (1.57)	0.81	0.438
rDNA_CpG_25.26	0.62 (0.79)	0.79	0.433	0.30 (1.15)	0.27	0.793	1.87 (1.21)	1.55	0.134	0.18 (1.62)	0.11	0.916
BNIP3L_Amplicon1_CpG_10	0.33 (0.83)	0.40	0.689	−0.17 (0.74)	−0.24	0.814	−0.26 (1.12)	−0.23	0.821	−1.15 (0.68)	−1.69	0.099
COX18_CpG_2	−0.01 (0.19)	−0.08	0.939	−0.18 (0.23)	−0.78	0.436	−0.35 (0.28)	−1.25	0.224	0.16 (0.22)	0.73	0.469
COX18_CpG_15	−0.11 (0.14)	−0.83	0.408	−0.04 (0.12)	−0.36	0.722	0.13 (0.15)	−0.85	0.404	−0.05 (0.10)	−0.55	0.587
GABARAP_Amplicon2_CpG_7.8	0.04 (0.09)	0.404	0.687	−0.14 (0.10)	−1.46	0.149	−0.08 (0.09)	−0.98	0.338	−0.03 (0.11)	−0.31	0.762
MARCH5_Amplicon1_CpG_2.3.4	0.08 (0.09)	0.89	0.377	0.05 (0.09)	0.63	0.533	0.00 (0.06)	0.03	0.980	−0.11 (0.06)	−1.729	0.092
RAB32_Amplicon1_CpG_24	−0.36 (0.30)	−1.18	0.240	0.21 (0.23)	0.88	0.381	**0.93 (0.39)**	**2.42**	**0.023**	0.14 (0.21)	0.66	0.514
RHOT2_Amplicon1_CpG_16	0.04 (0.28)	−0.13	0.896	0.33 (0.25)	1.34	0.183	−0.10 (0.29)	−0.36	0.723	0.13 (0.23)	0.59	0.558
TFB1M_CpG_12.13	−0.37 (0.42)	−0.90	0.375	−0.14 (0.31)	−0.46	0.647	−0.25 (0.46)	−0.54	0.594	−0.23 (0.29)	−0.79	0.436
GDMI	−0.30 (0.97)	−0.31	0.760	0.54 (1.04)	0.52	0.608	**−2.13 (1.05)**	**−2.02**	**0.052**	−0.79 (0.91)	−0.87	0.393

*Highly significative GDMI values are reported in bold*.

The analysis of specific sites showed that in most cases their methylation level was not associated with MNA. Among the few exceptions, rDNA_CpG_5 methylation levels which increased as MNA-SF scores also increased (*P* = 0.042). No significant correlations between MNA-SF and epigenetic biomarkers were detected in S2 group (Table 3).

Among the subjects who obtained a screening score lower than 12 in S1 group, we found that after adjusting for age at recruitment and gender, RAB32_Amplicon1_CpG_24 methylation levels were significantly correlated with the MNA-LF scores (*P* = 0.023).

After adjusting for multiple comparisons, none of the reported associations hold the statistical significance (Bonferroni corrected *p*-value 8.9 × 10^−^4). However, this correction seems more suitable when searching for associations without *a priori* hypotheses but too conservative when assessing a specific research question, as in our case, where a number of non-independent tests (due to the correlation between the analyzed markers) were also performed (59, 60).

## Discussion

This study reports the relationship between nutritional status, assessed by the MNA tool, a method extensively used to identify the risk of malnutrition in the elderly, and epigenetic biomarkers we previously identified associated to chronological and biological aging in human.

According to MNA analysis, the majority of the participants to our study was found to be well-nourished and, thus, their analysis was limited to the short form of the MNA tool. Our results indicate that four subjects out of five in the younger group and about half of the subjects in the older group have a good nutritional status. Malnutrition significantly influenced mortality risk in particular among the subjects belonging to the youngest age group where its effect was independent of the age at recruitment and the sex of the participants. The presence of an adequate nutritional status in subjects of the aged Calabrian population is also confirmed by the evidence that most subjects of both groups exhibit MNA mean scores higher than 17 that is widely considered the threshold of malnutrition. In our population sample, high prevalence of subjects who were well-nourished was observed. Probably this is related to the fact that the subjects enrolled in our study originate from a population where the social-economic context does not promote malnutrition and, in addition, the adoption of the Mediterranean diet may have provided an adequate and balanced intake of food. What is more all subjects we analyzed were home-care elderly so that it is likely that the good nutritional status reflects great attention by caregivers, in most cases family members, as suggested by Soini et al. (61).

Results here reported suggest that malnutrition is correlated with global DNA methylation status since low global DNA methylation levels (high GDMI values) are associated with malnutrition. This adds new data in the already well-demonstrated relationship between DNA methylation and frailty (8). In fact, a number of literature reports suggest that health outcomes attributed to malnutrition seem to be associated with frailty. Nevertheless, as emphasized by Wei et al., the natural history of malnutrition and frailty with respect to each other is still unclear, and indeed our data on specific sites indicate that many aspects of this relationship need to be clarified (62). This history is even more complex if we introduce the state of methylation that affects both. It is plausible to retain that malnutrition through epigenetic changes at global levels exacerbates the reduction of muscle, fat and bone mass observed during aging. This reduction contributes to sarcopenia and, in more severe form, to the decline of physical performance that ultimately may decrease the survival rate.

On the contrary, most of the sites located within specific genes, which were previously reported to be correlated with chronological and biological aging, showed to be not affected by malnutrition. Moreover, the few methylated sites associated with MNA scores showed correlations not immediately in line with previous results. Indeed, we found a direct correlation between a good nutritional status and the methylation levels of rDNA_CpG_5 and RAB32_Amplicon1_CpG_24, while we have previously reported that high proportion of the methylation of these sites is correlated with impaired cognitive performance, decreased survival chance and disability (11, 58). These results may suggest that malnutrition is among the first effects of disability or of other age-related specific problems (such as socio-economic problems, depression, or other) and then the initial response is a generalized non-specific epigenetic remodeling. By contrast, the fine remodeling of specific genomic sites is scarcely affected by malnutrition and may respond to a more complex interaction of different factors, such as metabolites correlated to senescence or to oxidative stress. This confirms that these biomarkers are very reliable in gauging the organism aging and are not misled by malnutrition (63).

The above associations are restricted to the subjects of S1 group (<85), possibly because malnutrition may need some time to act on methylation and the higher mortality of the subjects older than 85 years (S2 group) may lead to death very soon after the start of malnutrition without the time to establish a methylation remodeling of these sites.

Finally, it is likely that genetic variants impairing nutrient intake and/or metabolism which could have a role in the interplay we observed and create an additional layer over epigenetics changes.

We are aware that our study has some weaknesses that should be addressed. A first limitation of the study is the reduced sample size. Its cross-sectional design does not allow us to assess the cause-effect relationship between the variability of epigenetic markers and the nutritional status of subjects under study. Another important limitation is the lack of proper correction for multiple testing. However, since this study was exploratory and hypothesis-driven, a Bonferroni correction would have eliminated potentially important findings if applied (59, 60). For these reasons, further explorations in additional study populations are needed before conclusions can be drawn.

The finding of the influence of nutrition on frailty through epigenetic modifications appears particularly relevant because of a balanced nutritional intervention could be an easy to use clinical approach for population-based strategies in aging to provide favorable functional and mortality outcomes and, at the same time, minimize the hospital assistance and long-term care, thus reducing the health care costs.

## Data Availability Statement

The datasets generated for this study are available on request to the corresponding author.

## Author Contributions

PD'A, AM, GP, and DB designed the study. AM and VR performed the statistical analysis. DB and GP wrote the initial draft. AM, PD'A, VR, GP, and DB participated in critical revision and approved the final manuscript before submission.

### Conflict of Interest

The authors declare that the research was conducted in the absence of any commercial or financial relationships that could be construed as a potential conflict of interest.
