# Supplementary material for: Mini Nutritional Assessment Scores Indicate Higher Risk for Prospective Mortality and Contrasting Correlation With Age-Related Epigenetic Biomarkers
Source: Front Endocrinol (Lausanne). 2019 Oct 1;10:672. doi: 10.3389/fendo.2019.00672 (PMC6779723; doi:10.3389/fendo.2019.00672)
Supplement: Supplementary file 1 [file Table_1.DOCX]

rDNA gene promoter

GTCCTTGGGT TGACCAGAGG GACCC**CG**GG**C G**CTC**CG**TGTG TGGCTG**CG**AT 50

GGTGG**CG**TTT TTGGGGACAG GTGTC**CG**TGT C**CG**TGT**CGCG CG**T**CG**CCTGG 100

GC**CG**G**CG**G**CG** TGGT**CG**GTGA **CGCG**ACCTCC **CG**GCCC**CG**GG GGAGGTATAT 150

CTTT**CG**CTC**C G**AGT**CG**GCAA TTTTGGGC**CG** C**CG**GGTTATA TGCTGACA**CG** 200

CTGTCCTCTG G**CG**ACCTGT**C G**CTGGAGAGG TTGGGCCTC

BNIP3L

GGCTTGTTGT GTTGCTGCCT GAGTGC**CG**GA GA**CG**GTCCTG CTGCTGC**CG**C 50

AGTCCTGCCA GCTGTC**CG**AC AATGT**CG**TCC CACCTAGT**CG** AGC**CG**C**CG**C**C** 100

**G**CCCCTGCAC AACAACAACA ACAACTG**CG**A GGAAAATGAG CAGTCTCTGC 150

CCC**CG**C**CG**GC **CG**GCCTCAAC AGTGAGTG**CG** GGGC**CG**AGGC TCTGTGAAGG 200

GGATGGGGGA GGAGGAGCAG CCC**CG**GC**CG**C **CG**CCAC**CG**G**C GCG**G**CGCG**GG 250

AGG**CG**GGAGG AGAAGGCAGC TCATTGGCTC

COX18

GGATGTAGTG CTGGTAGGCT GCCAAAGGCA G**CG**TGACAGC ACCC**CG**TAAG 50

GCCA**CG**GTGG AGAGCAGAAT GCTGCCCCAC CAGGGCAGGC C**CG**TGG**CG**G**C** 100

**G**TGCA**CG**C**CG** AGCAGTACTT CCTC**CG**CAAC C**CG**CAC**CG**G**C G**AAGA**CGCG**G 150

CCAGGGCCT**C G**TACCAGC**CG** TT**CG**CATGTA CTGCAGAGAC TGGTGCCACT 200

GCCCACACTG GGAGAGTGGG G**CG**CTTGG**CG** C**CG**CT**CG**TAG GAAC**CG**G**CG**C 250

AAG**CG**GCAGG TCCCTAGCCC AAAGCTGCAG GGC

GABARAP

GTCCCCTCAA GGAAGCTGGG GCTGAAGAGG AGTAAAAGAT GGTAATCATC 50

ATA**CG**AGACT TGGTTCTCCA AGTTCCTTT**C G**TTAACAA**CG** TAGAGGAACA 100

GCAGGGACAA TTACAAGGTT AGCTATTCA**C G**AAC**CG**TGTT GCTA**CG**CTGA 150

AGG**CG**GC**CG**T TGACACCAAA ACAAAGTAGT ACCCAAGTGG **CG**GAGATGAT 200

CTCCAGAAAT AAGAAGTCAA AAAGAAAACA GATGTTTGGA GAGATCTACA 250

GAA**CG**CTTAA GTGCCAAAAT GAGTAGACCA ATAGGGACTG GAGAGGAGGT 300

TGGAAAGGTA GGGACTA**CG**C CAG**CGCG**GAG GAACACTG**CG** GGACTTGAGT 350

TAAATCATGT GATCTC

MARCH5

AGGTGGTGTA ATTCCCCCAA AATGGGCTCT GC**CG**CAGGAG AGGCTGGCTC 50

CAG**CG**C**CG**GG GGCTT**CG**GAA GGAGTTTCTG CCACCCCCAC TGC**CG**CCACT 100

GAC**CG**CCC**CG** A**CG**CCA**CG**GC **CG**GGGC**CG**GG GACCCTGATA AGAAATGGCC 200

CTTCAGCCCC CTCCCCTCAC CTGGCT**CG**GT CCCACCTGAG GGCAAGAG**CG** 250

GAGGCAAAAA CAAACAGGCA GGGAGGGCTG A

RAB32

GGGAGAGGAA GTCCAGCTGG GCC**CG**GC**CG**G GCTT**CG**GAGG **CG**C**CG**CC**CG**G 50

GAGAGGAAGT CCAGTTGGGC C**CG**GC**CG**GGC TTCAGAGG**CG** CAGGG**CG**GGA 100

GC**CG**CCT**CGC G**CAGGGTCCT CCCCAAGC**CG** G**CG**CCAGGCC CTGCCCT**CG**T 150

C**CG**GCCCTGC CCT**CG**TCTGG CCC**CG**CC**CG**G GC**CGCG**AGCA CTGG**CG**GGTT 200

CTGGGTCCTG TGAC**CG**GTCA GG**CG**G**CG**TCA G**CG**GG**CGCG**G **CG**GAGGGCTG 250

GC**CG**GCCT**CG** GGGGAGTTTC **CGCG**GC**CG**C**C G**GGGG**CGCG**G **CG**GCAGAG**CG** 300

**CG**AGGC**CG**GG CAGGGGGCCA GACT**CG**GAGT **CG**AGG**CGCG**C C**CG**ACAGC**CG** 350

CAG**CG**CTCAT GG**CG**GG**CG**GA GGAGC**CG**GGG ACCC**CG**GCCT GGGGG**CG**GC**C** 400

**G**C**CG**CCCCAG **CG**CC**CG**AGAC C**CGCG**AGCAC CTCTTCAAGG TGCTGGTGA 450

RHOT2

TGGAGTCTCT TTGTCCCCCT AGAAGC**CG**AG CAGA**CG**GA**CG** AGGAGCTG**CG** 50

GGAGGAGATC CACAAGGTAC C**CG**TGGTG**CG** **CG**GGA**CG**AGG GAGGGGCTGG 100

G**CGCG**GGCT**C G**GCCTAATC**C G**CTT**CG**CAGC CTGGGGGATT GGAC**CG**AGGT 150

GCTC**CG**GGTG TCCTTGGCCC TGATAATTCT GTGACCTC**CG** CACTGAGGGT 200

TGT**CG**GGGCC CCTACAG**CG**C ACCC**CG**CTGG GAGC**CG**GCAC **CG**CTCAGTCC 250

AGTGGTGCTC CAGGGATAAC AGGACCC

TFB1M

CCTCAAGTCC AGGAGGAAAT TCTGTGATAG CTGCTT**CG**CT GCTTGCAGTC 50

TTAACAACTT AATGATTTCT **CG**AAT**CG**TGG GCAA**CG**GAGG GAGA**CG**GCAA 100

GTGCTGAGTT TTC**CG**GAGGC AGCCATGATA **CGCG**GCAAGC ACCATCCAAC 150

CCTACCTCAC CCAGGACCTT CAC**CG**C**CG**CT C**CG**AAAGAAA **CGCG**CAGGGG 200

AGGAACCTG**C G**AGACCTAAG GCC**CG**CCT**CG** GAGTCAGCCC CATTGGTCAG 250

ACCTATCCCA C**CG**GAAG**CG**A TGAC**CGCG**GA CAGGAAATTC C**CG**G**CG**TGCT 300

GAGAG**CG**CAT G**CG**CTAAGTC CTGC**CGCG**AG AAGGGCAGGC TGGGTGGT**CG** 350

G**CG**GCTTC**CG** CCTGTGAGAG C**CG**GGGGAGA GC**CG**GGTGGA CTAGGCTTCT 400

CCTGG

**Figure S1.** Sequence of the genomic regions including all the analyzed CpGs (bold). The underlined CpG sites were found associated with MNA scores.
